# Association between subcutaneous and intramuscular fat content in porcine ham and loin depending on age, breed and *FABP3* and *LEPR* genes transcript abundance

**DOI:** 10.1007/s11033-012-2311-7

**Published:** 2012-11-29

**Authors:** M. Tyra, K. Ropka-Molik, A. Terman, K. Piórkowska, M. Oczkowicz, A. Bereta

**Affiliations:** 1 Department of Animal Genetics and Breeding, National Research Institute of Animal Production, Balice, Poland; 2 Laboratory of Genomics, National Research Institute of Animal Production, Krakowska 1, 32-083 Balice, Poland; 3 West Pomerian University of Technology, Piastów 17, 70-310 Szczecin, Poland

**Keywords:** Subcutaneous fat, Intramuscular fat, *FABP3*, *LEPR*, Gene expression, Pig

## Abstract

The objective of the present study was to analyze the level of intramuscular fat (IMF) in loin (*musculus longissimus dorsi*) and ham (*musculus semimembranosus*) and the level of subcutaneous fat in these cuts depending on breed, age and the expression level of *FABP3* and *LEPR* genes. The results obtained showed that only the breed influenced on the level of both intramuscular and subcutaneous fat to the same extent (*P* ≤ 0.001). The age of animals had an effect on fat content of the cuts (*P* ≤ 0.001) and to a lower extent on the level of IMF in both muscles (*P* ≤ 0.05). We confirmed highly significant effect of breed and age on the *LEPR* mRNA abundance—the expression of the this gene increased significantly (*P* ≤ 0.01) with age and the highest expression was found for the Puławska breed in *m. longissimus dorsi* and for the Polish Landrace breed in *m. semimembranosus.* We observed the high correlations between the transcript level of the *LEPR* gene and the fat content of individual cuts (*P* ≤ 0.01). The expression level of *FABP3* gene influenced the level of IMF (*P* ≤ 0.01), but not the level of subcutaneous fat in loin and ham.

## Introduction

It is well known that intramuscular fat (IMF) plays an important role both in the sensory evaluation of meat and meat products and in the suitability of meat for technological processing. In addition, to taste properties such as increased juiciness and tenderness, proper IMF levels prevent the loss of water during heat treatment (grilling, roasting or cooking) of meat. The direction of selection, which has been observed in recent years, results from efforts to eliminate the consequences of long-term marginalization of fatness traits due to selection pressure for increased carcass meatiness. This caused a considerable increase in the growth rate of pigs while having a negative effect in the form of lower IMF levels. In the Polish population of pigs, the level of IMF began to decrease at an alarming rate for the most numerous breeds (Polish Landrace and Polish Large White) [1, 2]. According to Schwab et al. [3], it is a result of genetic changes that have taken place in the populations being improved and the resultant proportions between the rate of adipose tissue and IMF deposition. On the other hand, even among high-producing pigs there are breeds characterized by rewarding IMF levels, as exemplified by the Duroc breed. In Polish breeding, there is one local breed (Puławska) characterized by a high (>2 %) level of intramuscular [2].

Current efforts are directed to find the genetic basis of variation of IMF content within the analysed populations and breeds as well as differences in the level of this trait between the breeds. Some positive results were obtained by research concerning the significance of leptin receptor (*LEPR*) and fatty acid binding proteins (FABP) in lipid metabolism. The proteins of this group, including H-FABP (heart fatty acid binding protein), transport fatty acids from cell membranes to their β-oxidation sites in the mitochondria [4], and due to this function *FABP3* gene is proposed asa candidate gene responsible for IMF levels in pigs. The *LEPR* gene coding for the long-form leptin receptor (Ob-Rb) is also considered as a candidate gene for QTL affecting carcass fatness in pigs [5]. The highest *LEPR* mRNA expression was found in the hypothalamus area which is associated with the regulation of feed intake and glucose homeostasis by leptin [6]. Furthermore, Vincent et al. [7] showed that in pigs, *LEPR* polymorphism affected daily gain and fat deposition.

In pigs, the search for closer relationships between polymorphic forms of *FABP3* and *LEPR* genes and the level of fatness, especially the level of IMF, was reported by the findings of Gerbens et al. [8, 9], Zhao et al. [10] and Li et al. [11]. Arnyasi et al. [12]. showed that *FABP3* polymorphism may account for 30–35 % of IMF variation in the population studied. However, the results obtained about the effect of *FABP3* gene polymorphisms on IMF level are not consistent. Gerbens et al. [13] found a relationship between the level of IMF and *FABP3*: c.1489C>T and c.1811G>C mutations only for barrows. On the other hand, Schwab et al. [14] and Tyra and Ropka-Molik [15] reported that the *FABP3:*c.103C>T mutation effected on IMF content and backfat thickness. Urban et al. [16] showed that this mutation has no effect on the level of fatness. Such inconsistent results may exclude the causal nature of the analysed mutations. Therefore, it was appropriate to analyse the effect of the transcript level of *FABP3* and *LEPR* genes on the level of fat traits in both the breed and age configurations.

A number of research confirm the lack of a common direction in accumulation of intramuscular and subcutaneous fat. The increase of subcutaneous fat of ham and loin, with age is probably due to physiological role of fat in the some processes such as reproduction, which is particularly manifest in gilts, i.e. in the sex investigated in our study. The role of IMF in the body is connected with the level of energy metabolism in the muscles, and it is necessary to find new sources of variation of these traits. Therefore, the aim of the present study was to analyse the level of IMF in loin (*musculus longissimus dorsi*) and ham (*musculus semimembranosus*) and the level of subcutaneous fat in these cuts depending on breed, age and the expression level of *FABP3* and *LEPR* genes.

## Materials and methods

We analyzed five pigs breeds: Duroc, Pietrain, Puławska (PUL), Polish Large White (PLW) and Polish Landrace (PL), raised in Poland and exhibit different fatness level including IMF content. The pig breeds examined in our study were included in the national breeding program as a maternal component (PLW and PL breeds) and paternal component (Duroc and Pietrain breeds). The Puławska breed represents an additional group which constitutes the genetic reserve. Animals belonging to this breed are kept locally and characterized by very good meat quality parameters, especially a high level of IMF content. All breeds were represented by 36 gilts. Animals were kept at the Pig Performance Testing Station (SKURTCh) in Pawłowice under the same housing and feeding conditions. According to the day of slaughter, animals of each breed were divided into six age groups (six sows per group): 60-, 90-, 120-, 150-, 180- and 210-day-old pigs. Sows of each breed were related, had the same father (except the Puławska breed, which had three fathers), and their mothers were sisters.

After 24 h chilling at 4 °C, right half-carcasses were dissected. All half-carcass components were weighed. The loin and ham were subjected to detailed dissection into individual tissues (lean, bone, subcutaneous fat, intermuscular fat, skin) to estimate fat percentage in carcass and cuts. The content of IMF in *m. longissimus dorsi* and in *m. semimembranosus* was also estimated in the study. All samples were taken from the middle area of muscle section except the samples of *m. longissimus dorsi*, which were taken from behind the last rib. IMF content of lean was determined as crude fat in a Soxhlet apparatus by extraction with fat solvents (Soxtherm SOX 406, Gerhardt).

Tissue fragments were collected immediately after slaughter. Samples were stored at −80 °C. The total RNA was isolated using TRI-Reagent (Sigma-Aldrich, Poznan, Poland). The concentration and quantification of RNA was estimated by spectrophotometer (Eppendorf, Hamburg, Germany) and by 2 % agarose gel electrophoresis. 1 μg of total RNA was transcribed into cDNA at 37 °C using a High Capacity cDNA Reverse Transcription Kit (Applied Biosystems, Warsaw, Poland), according to manufacturer’s protocol.

Relative quantification of the transcript level of *FABP3* and *LEPR* genes was quantified on 7500 Real-Time PCR System (Applied Biosystems). Relative Quantification for each gene was performed in multiplex reaction (in three repeats) with endogenous control—*GAPDH* gene. Primers and probes were described previously by Tyra et al. [17]. We ensured using controls without reverse transcriptase. Furthermore, the purity of RNA was checked by real-time PCR with “No-RT” control.

The real-time quantitative PCR analyses in a final volume of 25 μL contained: 12.5 μL TaqMan Universal PCR Master Mix, 0.5 μL of specific probes (250 nM final concentration), 0.5 μL of primers (900 nM final concentration) and 2.5 μL of cDNA. The results were analyzed using Sequence Detection System 7500 software v. 2.0.1 (Applied Biosystems).

The data were analyzed with the use of the GLM procedure (SAS Institute, Cary, NC, USA; v. 8.2, 2001). The final model was for general analyses:

$$ {\text{Y}}_{\text{ijklm}} = \mu + {\text{ d}}_{\text{i}} + {\text{ g}}_{\text{j}} + {\text{ f}}_{\text{k}} + \, \left( {\text{gf}} \right)_{\text{jk}} + {\text{ e}}_{\text{ijkl}} $$

where y_ijkl_ ijkl-th is the observation, μ the general mean, d_i_ the effect of i-th sire, g_j_ the effect of j-th breed, f_k_ the effect of k-th group of age, (gf)_jk_ the interaction between breed and age, e_ijkl_ the random error.

If the interaction was significance it was include in the model. Differences between the means for individual breeds were tested at 5 and 1 % levels using Scheffe’s multiple range test. Pearson’s correlations between the level of gene expression in relevant muscles and fatness traits of carcass and cuts and IMF content were estimated using the COR procedure of SAS software.

## Results and discussion

**Fig. 1:**
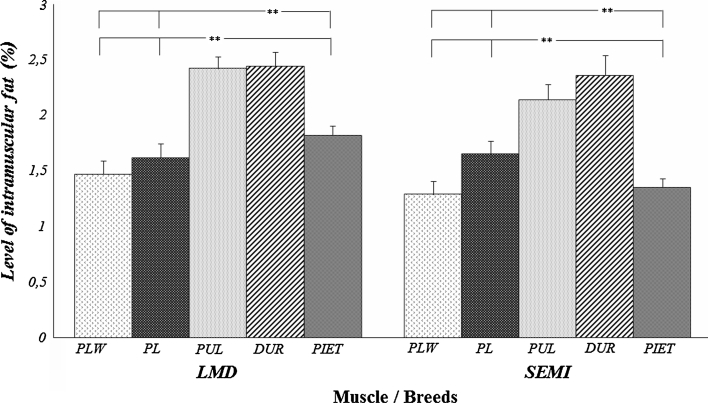
Level of intramuscular fat (%) in different muscles and breeds of pigs (*LMD m. longissimus dorsi*; *SEMI* *m. semimembranosus*; *PLW* polish large white; *PL* polish landrace; *PUL* puławska; **P < 0.01)

**Fig. 2:**
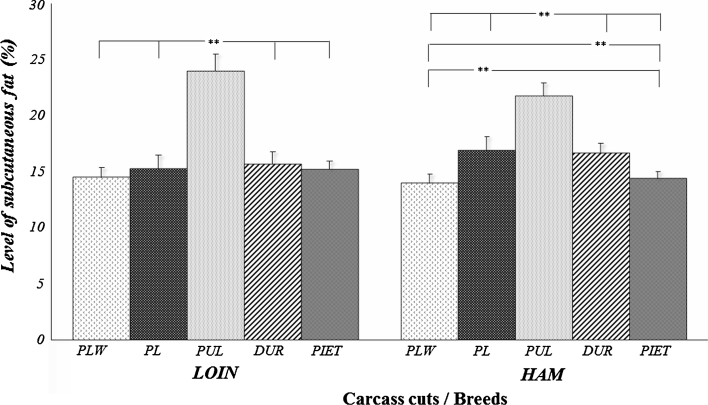
Level of subcutaneous fat (%) in different carcass cuts (loin and ham) and breeds of pigs. *PLW* polish large white; *PL* polish landrace; *PUL* puławska; **P < 0.01

In present study we analyzed the significance of factors (age, breed and expression of *FABP3* and *LEPR* genes) that may influence on the level of loin and ham fatness traits (Tables 1, 2). The analyzed parameters were the level of IMF in the loin (*m. longissimus dorsi*)—IMF_LMD_ and ham (*m. semimembranosus*)—IMF_SEMI_, and the level of subcutaneous fat in these cuts (SF_LMD_, SF_SEMI_). The mean content of IMF for all the test animals of different breeds and ages was below 2 % (1.95 % for *m. longissimus dorsi* and 1.76 % for *m. semimembranosus*). The level of subcutaneous fat in both carcass cuts was similar and approximated 17 %. The analysis that accounted for the effect of breed and age (Fig. 1; Table 3) showed that the level of IMF was significantly lower in *m. semimembranosus* compared to *m. longissimus dorsi*. No such differences were observed for the level of subcutaneous fat (Fig. 2; Table 4). Among the analysed factors, only the breed influenced on the level of both intramuscular and subcutaneous fat to the same extent (*P* ≤ 0.001). The age of animals had an effect on fat content of the cuts (*P* ≤ 0.001) and to a lower extent on the level of IMF in both muscles (*P* ≤ 0.05).

**Table 1:** Characteristics of the loin and ham fatness levels for selected pig breeds

Fatness traits	Means	±S.E.	Minimum	Maximum	*V*	GLM significance		
						Breed	Age	Breed × age
*IMF*_LMD_ (%)	**1.95**	*0.77*	0.29	4.06	39.5	***	***	***
*IMF*_SEMI_ (%)	**1.76**	*0.86*	0.22	5.21	49.2	***	*	*
*SF*_LOIN_ (%)	**16.98**	*7.41*	6.099	53.4	43.6	***	***	
*SF*_HAM_ (%)	**16.79**	*6.24*	6.30	38.8	37.2	***	***	

*n* = 180, *IMF* level of intramuscular fat in: *LMD m. longissimus dorsi*, *SEMI m. semimembranosus, SF* level of subcutaneous fat in loin and ham

* *P* < 0.05, *** *P* < 0.001, *S.E.* standard error (values in italics), values of means are bold

**Table 2:** Characteristics of the expression levels of *LEPR* and *FABP3* genes for selected pig breeds

Fatness traits	Means	±S.E.	Minimum	Maximum	*V*	GLM significance		
						Breed	Age	Breed × age
Gene expression								
*LEPR*_LMD_	**82.0**	*131*	0.17	657	159	***	***	***
*LEPR*_SEMI_	**37.2**	*57.1*	1.02	443	153	***	***	***
*FABP3*_LMD_	**2.53**	*1.52*	0.40	11.3	59.8	***		
*FABP3*_SEMI_	**3.96**	*2.49*	0.67	14.1	63.1	***		

*n* = 180, *LMD m. longissimus dorsi*, *SEMI m. semimembranosus*, *SF* level of subcutaneous fat in loin and ham

*** *P* < 0.001, the expression levels are presented as a relative quantity (RQ), *S.E.* standard error (values in italics), values of means are bold

**Table 3:** Level of intramuscular fat in muscles in different age ranges of pigs

Muscle	Age (days)	*n*	Means	±SD		Minimum	Maximum	*V*
*IMF*_LMD_ (%)	*60*	30	**1.70**	*0.98*	*Bdgi*	0.41	3.81	57.7
	*90*	30	**1.63**	*0.71*	*ACfh*	0.29	4.06	43.5
	*120*	30	**2.03**	*0.70*	*fg*	0.83	3.48	34.7
	*150*	30	**1.99**	*0.65*	*ehi*	0.72	3.95	32.7
	*180*	30	**2.26**	*0.64*	*AB*	1.04	3.48	28.2
	*210*	30	**2.11**	*0.76*	*Cde*	1.08	3.88	36.3
*IMF*_SEMI_ (%)	*60*	30	**1.85**	*0.81*		0.50	4.30	44.1
	*90*	30	**1.71**	*0.82*		0.22	3.63	47.9
	*120*	30	**1.51**	*0.69*	*A*	0.51	3.28	46.4
	*150*	30	**1.80**	*0.88*		0.92	4.76	49.2
	*180*	30	**2.06**	*1.17*	*Ab*	0.78	5.21	56.7
	*210*	30	**1.63**	*0.68*	*b*	0.62	3.13	42.3

*IMF* level of intramuscular fat in: LMD, *m. longissimus dorsi*; SEMI , *m. semimembranosus*

Values with the same letters indicate significant differences between group of age (A. B… = *P* ≤ 0.01; a. b … = *P* ≤ 0.05)

*SD* standard deviation (values in italics), values of means are bold

**Table 4:** Level of loin and ham fatness in different age ranges of pigs

Muscle	Age (days)	*n*	Means	±SD		Minimum	Maximum	*V*
*SF*_LOIN_ (%)	*60*	30	**12.4**	*2.95*	*AEj*	7.50	18.8	23.8
	*90*	30	**12.6**	*4.82*	*BFk*	6.09	23.2	38.1
	*120*	30	**14.3**	*4.51*	*CGl*	7.58	26.1	31.5
	*150*	30	**17.1**	*6.35*	*DHjkl*	6.11	30.1	37.2
	*180*	30	**21.7**	*6.87*	*EFGHi*	10.7	41.1	31.6
	*210*	30	**24.0**	*8.83*	*ABCDi*	12.6	53.4	36.8
*SF*_HAM_ (%)	*60*	30	**11.6**	*3.12*	*AEIL*	6.30	17.3	26.9
	*90*	30	**13.3**	*4.54*	*BFJ*	7.21	24.3	34.1
	*120*	30	**14.6**	*3.71*	*CGKL*	7.33	23.4	25.4
	*150*	30	**17.6**	*4.83*	*DHIJK*	9.25	29.9	27.5
	*180*	30	**21.1**	*6.03*	*EFGH*	12.1	38.8	28.6
	*210*	30	**22.7**	*6.04*	*ABCD*	13.6	38.6	26.6

Values with the same letters indicate significant differences between group of age (A. B… = *P* ≤ 0.01; a. b… = *P* ≤ 0.05)

*SF* level of subcutaneous fat in loin and ham

*SD* standard deviation (values in italics), values of means are bold

An analysis of impact of breed factor on IMF content in the muscles showed that Polish Large White, Pietrain and Polish Landrace pigs were characterized by lower IMF levels compared to the Duroc and Puławska breeds. The analysed breeds can be divided into three groups according to increasing subcutaneous fat levels in ham: Polish Large White and Pietrain (first group); Duroc and Polish Landrace (second group); and Puławska, (third group). Furthermore, Puławska pigs were the only animals with the highest ham fatness exceeding 21 % and this breed differ significantly (*P* ≤ 0.01) from the other in loin subcutaneous fat (24 %; Fig. 2). These results are consistent with the observations of a larger group of animals (over 4,500 pigs of all breeds raised in Poland) made during the monitoring of the national population, and with the results of the annual monitoring of gilt performance tests [2]. These authors showed an advantage of Duroc and Puławska animals in IMF levels and the highest level of carcass fatness in the Puławska breed. The dominant role of the Duroc breed among other high-producing breeds is also confirmed by other reports [18–21].

The Figs. 1 and 2 present the levels of ham and loin fatness characteristic of the analysed breeds. This results shows that there is no close relationship between the fat content of cuts and the level of IMF in the meat. The lack of this association is clearly demonstrated by the results obtained for Duroc and Puławska animals. These two breeds are similar in terms of IMF level but considerably different in the overall fat content of individual cuts and of the whole carcass [2]. Analogous observations were made by Suzuki et al. [22] and Cai et al. [23], who obtained low relationships between loin IMF content and overall carcass fatness (r_G_ = −0.03 and r_G_ = −0.13, respectively). This finding provides a possibility to carry out selection independently in both directions (increase of IMF content without concomitant increase in IMF).

The analysis of the effect of age on IMF content and level of ham and loin fatness, the results obtained showed that these two groups of traits are determined differently (Tables 3, 4). In the case of the fat content of cuts, both loin and ham fatness were found to increase as the animals grew older, and the differences between individual periods were mostly statistically significant (*P* ≤ 0.01). On the other hand, the lack of a directional relationship between age of animals and IMF level was found for *m. semimembranosus*. According to Schwab et al. [3], the unfavorable changes in the form of decreased rate of IMF deposition are the result of selection for a number of economically important traits. The rate of IMF deposition considerably slowed down, especially in relation to the rate of muscle tissue deposition. However, reaching the optimum IMF content would increase the final body weight while increasing the level of subcutaneous fat, which would be economically unfavorable [24]. These results support our conclusion about the lack of a direct relationship between the level of intramuscular and subcutaneous fat.

**Fig. 3:**
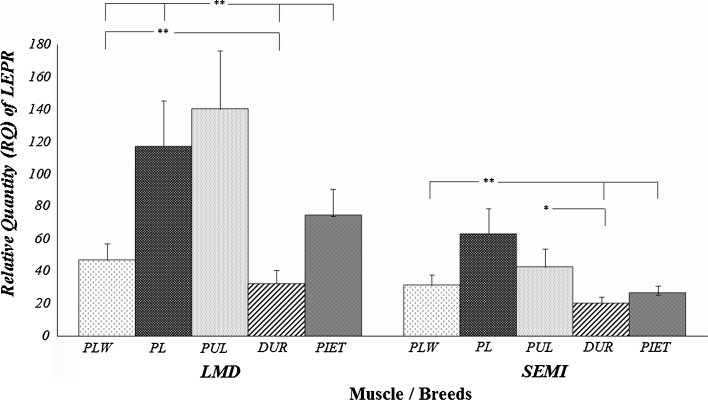
Relative quantity (RQ) of LEPR transcript in different muscles and breeds of pigs. *LMD m. longissimus dorsi*; *SEMI* *m. semimembranosus*; *PLW* polish large white; *PL* polish landrace; *PUL* puławska; *P < 0.05, **P < 0.01

**Fig. 4:**
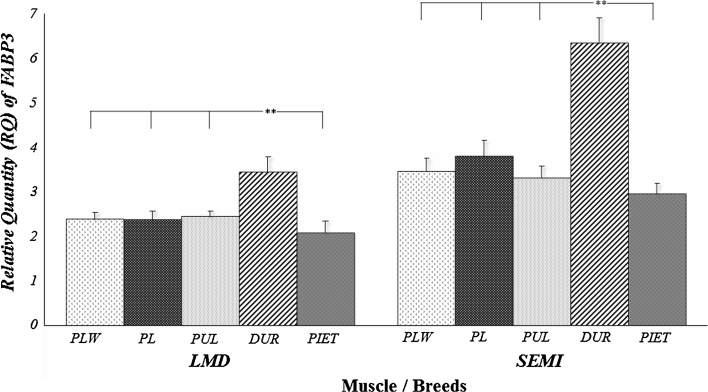
Relative quantity (RQ) of *FABP3* transcript in different muscles and breeds of pigs. *LMD m. longissimus dorsi*; *SEMI* *m. semimembranosus*; *PLW* polish large white; *PL* polish landrace; *PUL* puławska; **P < 0.01

Preliminary analysis performed for the expression level of *LEPR* and *FABP3* genes (Table 1) showed a highly significant effect of breed and age on the amount of transcript, but only for the *LEPR* gene. The expression level of the *LEPR* gene was higher in *m.* *longissimus dorsi* than in *m. semimembranosus,* which was also confirmed by other studies [17]. The analysis of impact of breed factor on the genes expression (Figs. 3, 4) showed a considerable advantage (*P* ≤ 0.01) of the Duroc breed for the *FABP3* gene in muscles analysed. The highest amount of the *LEPR* mRNA was found for the Puławska breed in *m. longissimus dorsi* and for the Polish Landrace breed in *m. semimembranosus*. In both cases, breed configuration did not fully match the fat content of cuts and the level of IMF in both muscles analyzed. Meanwhile, the advancing age of the animals was paralleled by a significant (*P* ≤ 0.01) increase in the transcript level of the *LEPR* gene in both muscles analysed (Table 5). The increase of expression level of *LEPR* gene translated into an analogous increase in the level of subcutaneous fat in both cuts studied (Table 4). This trend of the effect is also confirmed by the high correlations between the transcript level of the *LEPR* gene and the fat content of individual cuts, i.e. r_P_ = 0.628 for loin fatness and r_P_ = 0.553 for ham fatness. The transcript abundance of the *LEPR* gene had no effect on the IMF content in both muscles studied. A different situation occurred for the *FABP3* gene (Table 6) whose expression level in the analysed muscles influenced the level of IMF, with correlation coefficients of r_P_ = 0.365 for *m. longissimus dorsi* and r_P_ = 0.252 for *m. semimembranosus*. No relationship was found between the amount of transcript of the *FABP3* gene and the level of subcutaneous fat in loin and ham, and there were no differences in the expression level resulting from age between the animals.

**Table 5:** Level of *LEPR* gene expression in muscles in different age ranges of pigs

Gene_Muscle_	Age (days)	*n*	Means	±SD		Minimum	Maximum	*V*
*LEPR*_LMD_	*60*	30	**8.79**	*6.83*	*AFJ*	0.17	25.8	78.0
	*90*	30	**14.7**	*21.5*	*BGk*	0.68	115	145
	*120*	30	**27.7**	*26.1*	*CHl*	1.78	109	94.1
	*150*	30	**76.1**	*73.2*	*DIJkl*	3.51	293	96.1
	*180*	30	**156**	*161*	*EFGHI*	12.6	575	103
	*210*	30	**223**	*195*	*ABCDE*	3.31	657	87.7
*LEPR*_SEMI_	*60*	30	**16.6**	*19.5*	*B*	1.67	68.5	118
	*90*	30	**17.2**	*22.9*	*C*	1.02	88.8	133
	*120*	30	**24.4**	*20.5*	*D*	2.02	68.9	84.3
	*150*	30	**34.5**	*38.7*	*E*	1.05	163	112
	*180*	30	**42.2**	*51.9*	*F*	2.99	215	123
	*210*	30	**91.8**	*106*	*ABCDEF*	7.22	443	115

Values with the same letters indicate significant differences between group of age (A. B… = *P* ≤ 0.01; a. b … = *P* ≤ 0.05), the expression levels are presented as a relative quantity (RQ)

*LMD m. longissimus dorsi*; *SEMI m. semimembranosus,* *SD* standard deviation (values in italics), values of means are bold

**Table 6:** Correlations between expression levels of *LEPR* and *FABP3* genes in different tissues and some parameters of loin and ham fatness

Traits of fatness	Gene_(Muscle)_			
	*FABP3*_(LMD)_	*FABP3*_(SEMI)_	*LEPR*_(LMD)_	*LEPR*_(SEMI)_
*IMF*_LMD_	0.365^**^	–	0.096	–
*IMF*_SEMI_	–	0.252^**^	–	0.039
*SF*_LOIN_	0.052	–	0.628^**^	–
*SF*_HAM_	–	0.120	–	0.553^**^

*IMF* level of intramuscular fat in; *LMD m. longissimus dorsi*; *SEMI m. semimembranosus*; *SF* level of subcutaneous fat in loin and ham

**P < 0.01

Li et al. [25] investigated differences in the transcript abundance of 90 genes associated with lipid metabolism between the Landrace and Taihu breeds during the first 5 months of life. The authors found statistically significant difference in the expression level of 25 genes including *FABP3* in adipose tissue. According to the same authors, the more intensive metabolism in the Landrace breed during this important stage of pig growth has an effect on the lower capacity to deposit IMF. The level of IMF in 5-month-old Landrace animals was 2.0 % compared to 4.4 % in the Taihu breed. Despite numerous reports about the high level of *FABP3* expression in the cardiac muscle and in skeletal muscles [26, 27], Li et al. [28] showed high amounts of *FABP3* mRNA also in the adipose tissue of adult pigs (about 30 % of the level found in skeletal muscles). The authors suggested to change the previously held view about the role of adipose tissue as a passive repository for energy to that of an important, active endocrine organ. This is all the more justified because of a large number of proteins and hormones secreted in white adipocytes [29]. Li et al. [11] estimated the expression level of the *FABP3* gene in skeletal muscles and subcutaneous fat in two groups of pigs with low (1.27 %) and high (3.32 %) levels of IMF. According to the authors higher level of mRNA was in the muscles of animals with the higher IMF levels. Similary to our research, Gardan et al. [30] found no differences in the *FABP3* mRNA level depending on the age of pigs (80 and 210 days). The factor that caused statistically significant differences in the transcript level of this gene was the type of fat in which the expression was studied, with higher expression observed in intramuscular compared to intermuscular fat. These results prove the complexity of the effect of carcass fatness parameters and of the not completely understood the role of *FABP3*, *LEPR* genes in this process. The complex nature of the lipogenesis process is evidenced by the fact that over 900 genes are candidate genes as QTL for fatness traits [31]. Such a large number of genes that determine fatness traits have prevented finding of one factor characterized by a large effect in this regard. However, research should be continued because beneficial changes may be obtained even with a small transfer of the genetic effect, aided by modern methods of breeding (genetic) value estimation, onto the whole population being improved.

## Conclusions

Both forms of fatness analysed in the present study, i.e. the level of subcutaneous fat and the level of IMF in both muscles (*m. semimembranosus* and *m. longissimus dorsi*) are probably determined by different genetic factors. This is evidenced by the correlations obtained between fatness traits and the expression level of *FABP3* and *LEPR* genes. At the same time, the level of subcutaneous fat does not directly determine the level of IMF. This finding is beneficial to breeders because it enables selection to be carried out independently in both directions (increasing IMF level and decreasing the level of subcutaneous fat). It has been confirmed that the age of the analysed animals has a large effect on the level of subcutaneous fat for both carcass cuts, with loin being characterized by a slightly higher rate of fat increase with age. This effect was not observed for IMF in *m. semimembranosus*, whereas in *m. longissimus dorsi* the effect of age was noticeable but when the observation period was longer, which is due to the decreasing rate of IMF deposition. It is concluded from the results obtained that despite the lack of direct relationships between these two forms of fat and with the present rate of their deposition in pig carcasses (especially their proportions) it is not possible for a pig population to produce carcasses characterized by a high level of IMF and a low level of subcutaneous fat. Therefore, it is important to find a factor that determines the rate of IMF deposition and apply it in the selection of pigs.
